# Deposit Plates

**Published:** 1891-10

**Authors:** 


### DEPOSIT PLATES.

It will be noticed by reference to our advertising pages that Dr. C. S. Stockton has assumed the charge of making these plates. From Dr. Stockton's well-known ability, we have no doubt that many of the difficulties incident to this work will be overcome, and they be made entirely satisfactory.

